# Establishment of a novel cellular model for myxofibrosarcoma heterogeneity

**DOI:** 10.1038/srep44700

**Published:** 2017-03-17

**Authors:** Birgit Lohberger, Nicole Stuendl, Andreas Leithner, Beate Rinner, Stefan Sauer, Karl Kashofer, Bernadette Liegl-Atzwanger

**Affiliations:** 1 Department of Orthopaedic Surgery, Medical University of Graz, Graz, Austria; 2 Biomedical Research, Medical University of Graz, Graz, Austria; 3 Institute of Pathology, Medical University of Graz, Graz, Austria

## Abstract

Human cancers frequently display substantial intra-tumoural heterogeneity in virtually all distinguishable phenotypic features, such as cellular morphology, gene expression, and metastatic potential. In order to investigate tumour heterogeneity in myxofibrosarcoma, we established a novel myxofibrosarcoma cell line with two well defined sub-clones named *MUG-Myx2a* and *MUG-Myx2b.* The parental tumour tissue and both MUG-Myx2 cell lines showed the same STR profile. The fact that *MUG-Myx2a* showed higher proliferation activity, faster migration and enhanced tumourigenicity was of particular interest. NGS mutation analysis revealed corresponding mutations in the FGFR3, KIT, KDR and TP53 genes. In contrast, the *MUG-Myx2a* cell lines showed an additional PTEN mutation. Analysis of CNV uncovered a highly aberrant karyotype with frequent losses and gains in the tumour sample. The two *MUG-Myx2* cell lines share several CNV features of the tumour tissue, while some CNVs are present only in the two cell lines. Furthermore, certain CNV gains and losses that are exclusive to either *MUG-Myx2a* or *MUG-Myx2b*, distinguish the two cell lines. As it is currently not possible to purchase two different sarcoma cell lines derived from the same patient, the novel myxofibrosarcoma cell lines *MUG-Myx2a* and *MUG-Myx2b* will be useful tools to study pathogenesis, tumour heterogeneity and treatment options.

Myxofibrosarcoma (MFS) is the most common sarcoma in elderly patients and is characterised histologically by a multinodular growth pattern and variably prominent myxoid stroma. The tumour is mainly composed of spindle cells with variable cytologic atypia accentuated along curvilinear vessels^1^. Clinically, increasing grades and stages of the tumours are frequently seen in MFS after local recurrences, which may eventually lead to metastatic diseases. Recurrence has been shown to occur despite repeated surgery involving wide-ranging local excisions and negative surgical margins^1^,^2^,^3^. MFS is characterized by non-specific cytogenetic aberrations that increase with grade, suggesting a multistep tumour progression model due to acquired genetic instability^4^. The mechanisms responsible for the coexistence of distinct sub-clones and the biological consequences of this coexistence remain poorly understood. MALDI imaging mass spectrometry demonstrated a high grade of intra-tumour heterogeneity in MFS^5^. Despite their histological resemblance, imaging mass spectrometry demonstrated that intra-tumour heterogeneity was a consistent feature in each of the MFS tumours studied, and revealed that different nodules exhibited high grade-/low grade-like biomolecular signatures. Hence, cytogenetic heterogeneity and clonal evolution seem to be common in myxoid sarcoma^5^,^6^. In addition, large-scale sequencing analyses of other solid cancers have identified extensive heterogeneity both between individual tumors and genetic intra-tumour heterogeneity^7^,^8^,^9^. Genomic instability generates a high level of intercellular genetic heterogeneity and has been linked to both drug resistance and poor prognosis in cancer, because therapeutic procedures rely on single tumour biopsy samples^10^,^11^,^12^.

In this context the availability of appropriate *in vitro* cell systems is of particular importance. Permanent cell lines derived from primary sarcomas offer the opportunity to study functional alterations in sarcoma biology. However, the supply of commercial sarcoma cell lines is extremely inadequat. In order to investigate this intra-tumour heterogeneity of MFS *in vitro*, we established two sub-clones of a primary MFS, named *MUG-Myx2a* and *MUG-Myx2b*. The new cell lines provide a model for tumour heterogeneity and allow comprehensive genetic and epigenetic characterization. Furthermore, because of their tumourigenicity, the cell lines are also suitable for *in vivo* experiments. The present study describes the establishment, various growth kinetics, migration activity, tumourigenicity, cytogenetic features and mutation analysis of the newly established MFS cell lines.

## Results

### Establishment of the novel MFS sub-clones *MUG-Myx2a* and *MUG-Myx2b*

**Figure 1:**
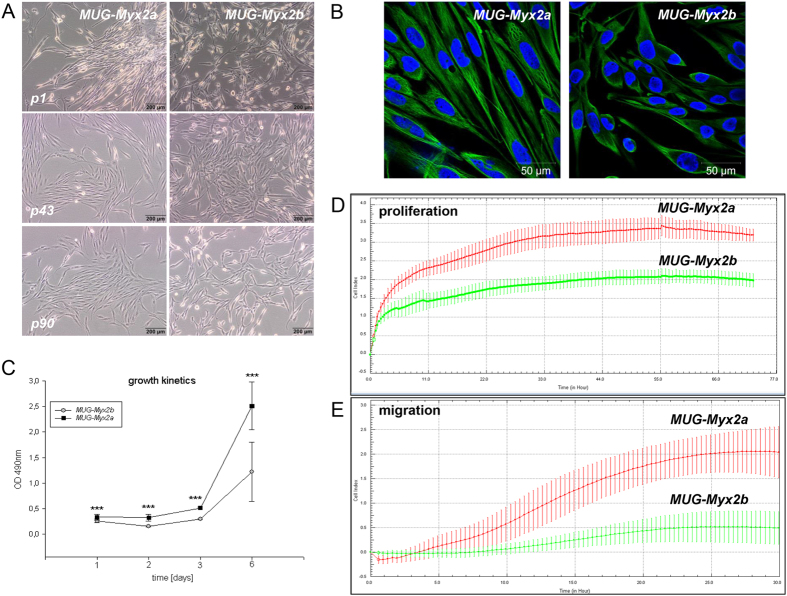
Establishment and characterization of the myxofibrosarcoma cell lines *MUG-Myx2a* and *MUG-Myx2b*. (**A**) Morphological features of *MUG-Myx2a* and *MUG-Myx2b* cells at passage 1, 43, and 90. (**B**) Strong expression of Vimentin of *MUG-Myx2a* and *MUG-Myx2b* confirmed the mesenchymal origin of the tumour cell lines; nuclei were counterstained with DAPI. (**C**) MTS proliferation analysis revealed highly significant variations at all time points. Dynamic proliferation curves measured with the xCELLigence system showed (**D**) a faster growth rate and (**E**) a higher migration potential for *MUG-Myx2a* cells than the *MUG-Myx2b* cells. X axis represents CI (cell index) and y axis the time in hours.

Haematoxylin and eosin (HE)-stained slides of a 94-year old female patient revealed a G3 stage MFS tumour. The tumour was composed of morphologically heterogenous areas. Partially, the tumour showed abundant myxoid stroma with classic curvilinear tumour vessels. In addition, accentuated atypical tumour cells with spindle morphology were located around vascular structures, consistent with well differentiated tumour areas. Furthermore, the tumour showed a high grade tumour component composed of fascicles of pleomorphic tumour cells and brisk mitotic activity. Immunohistochemical (IHC) staining of the patient’s tumour revealed only focal SMA positivity, whereas the tumour was negative for Desmin, Caldesmon, S100, CD34, EMA, Melan A, and Pan-CK. (data not shown). After crushing and enzymatically digesting the tumour tissue, starting from the primary culture (passage 0) two different sub-clones were successfully expanded. As early as the beginning of cultivation, the clones named *MUG-Myx2a* and *MUG-Myx2b* differed in terms of their morphology. *MUG-Myx2a* and *MUG-Myx2b* showed elongated spindle-shaped cells, with large nuclei and prominent nucleoli, and grown attached to a substrate. It is notable, that throughout the entire duration of cultivation *MUG-Myx2a* cells were markedly more elongated and more spindle-shaped than *MUG-Myx2b* (Fig. 1A, B). The mesenchymal origin of the cells was confirmed by high vimentin expression (Fig. 1B). To demonstrate the identical origin of the cell lines, a short tandem repeat (STR) Power Plex^®^ 16 analysis of the frozen primary parental tumour tissue, as well as *MUG-Myx2a* (passage 45) and *MUG-Myx2b* (passage 48) was performed. The original tissue and cell lines showed the same STR profile for the markers TH01, D21S11, Penta E, D5S818, D13S317, D7S820, CSF1PO, Penta D, Amelogenin, D8S1179, TPOX, and FGA. All values are summarised in Table 1.

### *MUG-Myx2a* cells show higher proliferation, migration, and tumourigenicity

**Figure 2:**
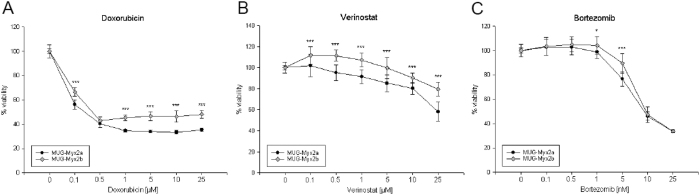
Analysis of the cytotoxic effect of chemotherapeutic agents on *MUG-Myx2a* and *MUG-Myx2b* cells. Both sub-clones were treated with (**A**) 0–25 μM doxorubicin, (**B**) 0–25 μM Verinostat, and (**C**) 0–25 nM Bortezomib and measured after 48 h.

The growth behaviour of the two clones was investigated with the MTS assay after 1 to 6 days. The statistical comparability of the two clones revealed highly significant variations at all time points (day 1: p = 0.00064; day 2: p = 1.87E-06; day 3: 2.82E-17; day 6: 6.23E-06; n = 12) (Fig. 1C). In order to evaluate the real-time growth pattern and migration potential, cells were detected in quadruplicate with the xCELLigence System. Using the RTCA 1.2.1 software, the population doubling time of the cells was calculated. *MUG-Myx2a* cells displayed a lower doubling time (20 h vs 22 h) and a higher migration potential than *MUG-Myx2b* cells (Fig. 1D,E). After treatment with 0–25 μM doxorubicin, 0–25 μM Verinostat, and 0–25 nM bortezomib for 48 h, cell viability was measured by the MTS assay. Chemotherapeutic drugs inhibited cell growth in a dose-dependent manner in both cell lines (Fig. 2A–C). Nevertheless *MUG-Myx2a* cells showed a significant higher sensitivity to all three therapeutic drugs.

**Figure 3:**
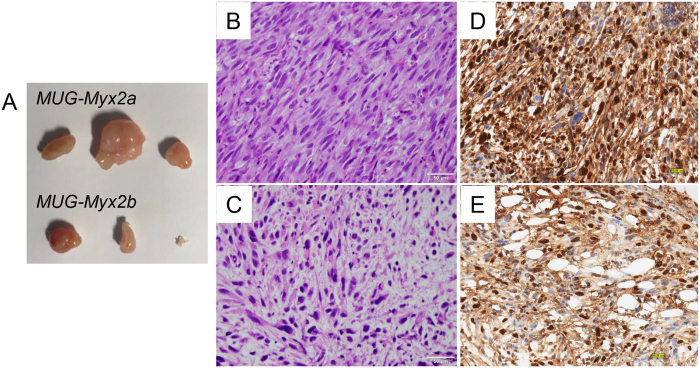
(**A**) *MUG-Myx2a* and *MUG-Myx2b* tumour formation in mice. (**B,C**) HE-stained FFPE mice tumours showed the variation concerning the morphology. (**D,E**) IHC of the proliferation marker Ki-67. *MUG-Myx2a* tumours from all mice displayed an increased proliferation level as compared to *MUG-Myx2b* tumours (n = 3).

*MUG-Myx2a* (p49) and *MUG-Myx2b* (p52) cells successfully formed tumours in 3 of 5 transplanted mice. Small nodules were palpable 4 weeks after inoculation. After 8 weeks the animal were dissected. Tumour formation in mice was significantly higher when *MUG-Myx2a* cells were injected, in comparison to *MUG-Myx2b* (Fig. 3A). The success rate of growing *MUG-Myx2a/b* cells in nu/nu Foxn1 mice was 60%. Successful engraftment was determined by pathological examination of formalin-fixed, paraffin-embedded (FFPE) material from the tumour samples. Hematoxylin and eosin (HE)-stained FFPE mice tumours showed variation concerning the morphology of *MUG-Myx2a* (Fig. 3B) and *MUG-Myx2b* (Fig. 3C). In particular, *MUG-Myx2b* showed the characteristic prominent myxoid stroma of MFS.

The high mitotic rate and high proliferative index were confirmed by IHC with the proliferation marker Ki-67. Using the ImageScope software, Ki-67 positive and negative cells were quantified after IHC staining. *MUG-Myx2a* tumours from all mice displayed an increased proliferation level, compared to *MUG-Myx2b* tumours (Ki-67 positivity: 0.937 ± 0.033 vs 0.604 ± 0.185; n = 3). A representative staining of one pair of tumours is shown and summarized in Fig. 3D (*MUG-Myx2a)* and 3E (*MUG-Myx2b)*.

### Mutation Analysis by Next-Generation Sequencing (NGS)

Mutation Analysis of 50 genes by NGS revealed that the primary patient’s tumour tissue and *MUG-Myx2b* showed identical mutations in the FGFR3, KIT, KDR, and TP53 genes. Conversely, the *MUG-Myx2a* cell line showed the same mutations as *MUG-Myx2b* and the primary patient tumour, but had an additional mutation in the PTEN gene (Table 2).

### Chromosomal Copy Number Variations (CNVs)

**Figure 4:**
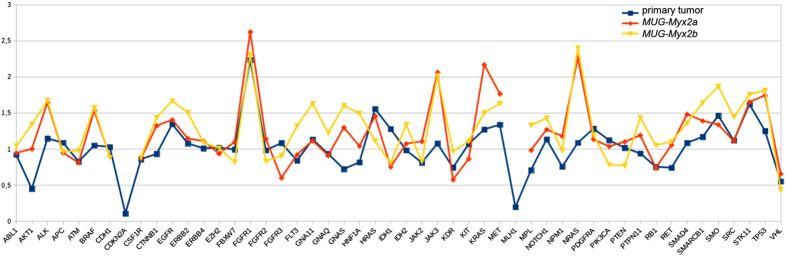
Circos plot showing CNVs across the whole genome from the patient primary tumour (outer ring) versus *MUG-Myx2a* (middle ring), and *MUG-Myx2b* (innermost ring) cell lines. red = loss, blue = gain.

**Figure 5:**
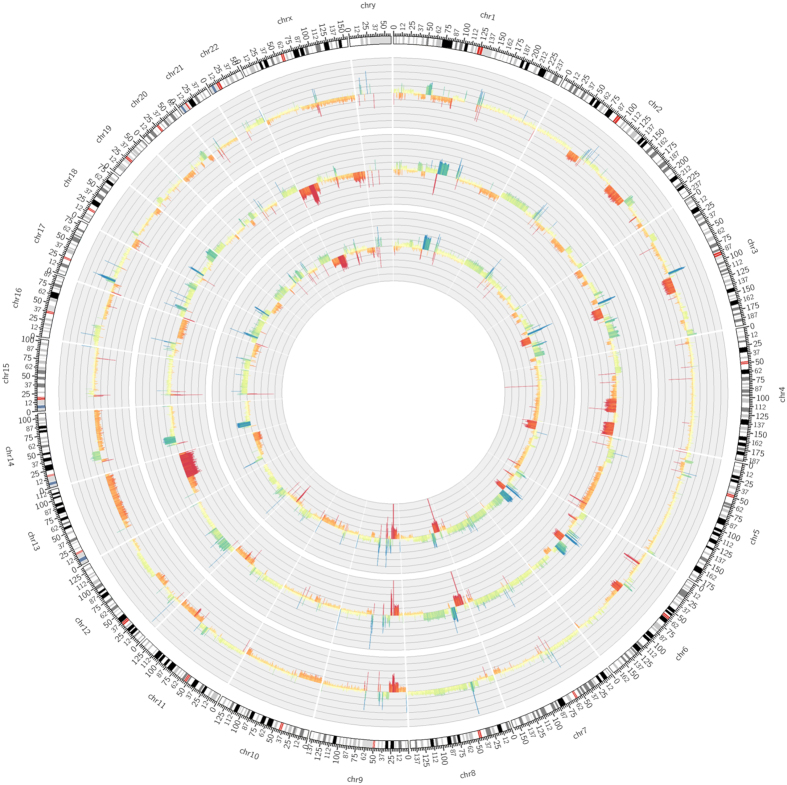
Gain and loss at chromosomal regions of 50 cancer genes in the patient’s primary tumour (blue) versus *MUG-Myx2a* (red), and *MUG-Myx2b* (yellow) cell lines. Y-axis represents ratio of chromosome ploidy centered on 2n.

Low coverage whole genome sequencing was used to determine chromosomal gains and losses in the primary tumour tissue as well as in both *MUG-Myx2* cell lines. The tumour tissue showed widespread gains and losses for several chromosomes (Fig. 4, outer ring). The *MUG-Myx2* cell lines both showed gains for chromosomes 3, 6, 8, 14, and 17 as well as losses of chromosomes 3, 6, 9, and 13. Additionally, both cell lines showed gains for chromosomes 1, 3, and 6 and losses of chromosomes 8 and X. These alterations were not evident in the primary tumour tissue. *MUG-Myx2a* (Fig. 4, middle ring) showed losses of chromosome 4 and gains of chromosome 12, but these characteristics were not shared with the primary tumour, or the *MUG-Myx2b* cell line. On the other hand, *MUG-Myx2b* (Fig. 4, innermost ring) exhibited losses of chromosome 5, which were exclusive to this cell line. In addition to these substantial CNVs, we also determined the copy number state of 50 cancer genes, which were also tested for the presence of mutations. Both *MUG-Myx2* cell lines showed a similar pattern of CNVs in these genes and shared the distinct loss of CDKN2a and MLH1, as well as a gain of FGFR1, with the primary tumor tissue (Fig. 5; Table 3). However, gain of the JAK3 and NRAS locus was only seen in the *MUG-Myx2* cell lines.

## Discussion

Myxofibrosarcoma is a malignant neoplasm with variably prominent myxoid stroma, cellular pleomorphism, and a distinct curvilinear vascular pattern. It represents the most common sarcoma in elderly patients, with a slight predominance in males^1^. In order to enable the investigation of tumour heterogeneity on cellular and molecular level, it is highly desirable to establish new human primary cell lines.

Clonal heterogeneity may be evident within single samples, but can also be observed between different tumour regions within the same primary site, or even between primary and metastatic sites, so-called “regional heterogeneity”^13^,^14^.

*MUG-Myx2a* and *MUG-Myx2b* cell lines showed the same STR profile and could be maintained in long-term cultures with a 20 or 22 h doubling time, respectively. Interestingly, the *MUG-Myx2a* showed a higher proliferation and migration ability than *MUG-Myx2b*. Additionally, compared to *MUG-Myx2b, MUG-Myx2a* cells showed a higher sensitivity to commonly used chemotherapeutic drugs.

Both cell lines successfully formed tumours in nu/nu Foxn1 mice, although the *MUG-Myx2a* cell line again showed higher proliferation capacity and thereby confirmed the growth analysis data. A nu/nu Foxn1 mouse model demonstrated, that both cell lines showed equally aggressive *in vivo* growth behaviour. Using an alternative mouse model, such asNSG mice, might show increased tumourigenicity.

A distinctive feature of cancer cells compared to normal cells is chromosome instability, which is proposed to be critical for the initiation of tumourigenesis^15^. For this reason, we investigated the genomic integrity of the *MUG-Myx2* cell lines by low density whole genome sequencing. Gains in gene copy number can drive the expression of oncogenes, whereas decreased gene dosage by hemizygous and/or homozygous deletion may inactivate tumour suppressor genes^16^. The *MUG-Myx2* cell lines match some, but not all chromosomal aberrations of the primary tumour. Interestingly, many features seem to be more pronounced in the cell lines, consistant with the notion, that the primary tumour contains several sub-clonal populations of cells which cumulatively contribute to the CNV phenotype of the primary tumour. However, the two cell lines might represent single sub-clones which share many features with the primary tumour, but have also accrued additional changes. In the CNV analysis of individual genes, the copy number state of the CDKN2a, MLH1, and FGFR1 genes is identical in the primary tumour and the cell lines. CDKN2a, also known as p16, has been described as frequently deleted in osteosarcomas^17^ whereas we found changes which are present in both the primary tumour and *MUG-Myx2a/b*. MLH1 is part of the DNA mismatch repair system and its loss could contribute to the widespread genetic changes seen in this tumour. FGFR1 amplification is a common feature of several tumour types and activates the cell cycle via the RAS pathway.

The recent development of NGS as a diagnostic tool in the clinical setting has enabled rapid, targeted sequencing of tumours to determine causative mutations. When combined with various selective capture approaches, NGS has allowed for the efficient simultaneous genetic analysis of a large number of candidate genes. Here, we applied polymerase chain reaction (PCR) based NGS to identify alterations to oncogenes and tumour suppressor genes. Our NGS data revealed, that both *MUG-Myx2a* and *MUG-Myx2b* cells possessed the p53 mutation R213Q, whereas, only *MUG-Myx2a* showed the PTEN R173H mutation.

p53 and PTEN are the two most frequently mutated tumour suppressors in human cancer. p53 plays a major role in regulating the response of mammalian cells to stress and damage, partially through transcriptional activation of genes involved in cell cycle control, DNA repair, senescence, angiogenesis, and apoptosis^18^. Furthermore, mutations in p53 have also been identified as the most common genetic alterations in sarcoma^19^. PTEN is involved in basic cellular functions including adhesion, migration, proliferation and cell survival^20^. Somatic mutations in PTEN are now known to cause tumourigenesis in a number of human tissues^21^. Both p53 and PTEN are involved in sustaining cellular homeostasis and complex regulatory interactions^22^,^23^. The combined inactivation pf p53 and PTEN could greatly accelerate tumour development^24^,^25^,^26^. Mutational analysis of p53 and PTEN in STS tissue samples revealed an incidence of 25.6% (22 out of 86) p53 mutations but only 2.3% (2 out of 86) PTEN mutations^27^. The PTEN R173H mutation is located in one of the three hotspot sites of the PTEN gene and has been reported in patients with PTEN associated tumors^28^. This mutation reduces PTEN activity by 50%^29^. The absence of this mutation from the primary tumor as well as the MUG-Myx2b cell line can either be explained by tumor heterogeneity, with MUG-Myx2a cells originating from a tumor sub-clone not present in the paraffin block sample used for analysis of the primary tumor, or by a secondary mutation during cell culture.

KDR (kinase insert domain receptor) is the human gene encoding vascular endothelial growth factor receptor 2 (VEGFR-2). It functions as the main mediator of VEGF-induced endothelial proliferation, survival, migration, tubular morphogenesis, and sprouting. Several studies have reported the genetic polymorphism of the KDR gene and the implicated risk of coronary artery diseases^30^,^31^. However, the clear role of individual KDR SNPs and their physiological functions in cancer progression and prognosis remains unknown. Mutations in the KIT receptor tyrosine kinase, which are commonly present in gastrointestinal stromal tumours (GISTs; 70–80% of all cases) are clustered in four exons^32^. The FGFR3 F386L polymorphism has been reported in association with low-grade tumours and early disease stage in prostate cancer^33^.

In conclusion, the well-characterized novel MFS cell lines *MUG-Myx2a* and *MUG-Myx2b* will be a useful tool to gain further insights into the pathogenesis and tumour heterogeneity of MFS and explore new therapy options.

## Methods

### Patient history

A 94-year-old Caucasian woman presented in May 2014, at the Department of Orthopedic Surgery at the Medical University of Graz with a soft tissue lump. Radiography and magnetic resonance imaging (MRI) revealed a tumour mass with an extent of 5 × 3.5 × 4.5 cm on the left knee. After a biopsy a wide resection was performed at our department and the knee joint was covered with a gastrocnemius muscle flap. The postoperative histopathological report revealed a MFS G3 with resection margins free of disease. The patient received adjuvant radiotherapy. The research reported in this study was conducted adhering to the principles of human welfare according to the Consort declaration on clinical research design and the Helsinki declaration on medical protocols and ethics. The study protocol and the informed consent of the patients were approved by the ethics committee of the Medical University Graz (vote #27–258ex14/15; valid until 17.04.2016). The patient was extensively informed and gave her written approval.

### Cell culture procedures

The tumour tissue was obtained immediately after surgical removal. After mechanical disaggregation of the tumour tissue into 1–2 mm^3^ pieces, the minced tissue was enzymatically digested with 2 mg/ml collagenase B (Roche Diagnostics, Mannheim, Germany) for approximately 20 h under constant rotation at 37˚C. After centrifugation at 1400 rpm for 5 min two fractions were isolated. On the one hand, the cell pellet was washed twice with PBS and plated in Dulbecco’s-modified Eagle’s medium (DMEM-F12; Invitrogen, Darmstadt, Germany), containing 10% foetal bovine serum (FBS), 1% L-glutamine, 100 units/ml penicillin, 100 μg/ml streptomycin and 0.25 μg amphotericin B (all Invitrogen). From this fraction the sub-clone *MUG-Myx2b* was grown. On the other hand, the viscous colloidal supernatant was collected and cultured as sub-clone *MUG-Myx2a* in the above-mentioned culture medium. During cultivation time, the cells were regularly cryopreserved. Cells grew to be adherent as a monolayer and were passaged more than 70 times during the period of 24 months. Even from the beginning of cultivation *MUG-Myx2a* and *MUG-Myx2b* differed regarding their morphology and growth behaviour (see Fig. 1). All cell cultures were periodically checked for mycoplasma by PCR.

### Cell line identification Power Plex^®^ 16 System

Frozen tumour tissue from the patient was dissected into small pieces and re-suspended in 180 μl ATL buffer (Qiagen, Hilden, Germany). Cell pellets (5 × 10^5^) from both clones (passage 45 and passage 48) were re-suspended in 200 μl PBS; subsequently 20 μl Proteinase K and 200 μl AL Buffer (Qiagen) were added. DNA preparations were performed using the QIAamp DNA Mini kit (Qiagen) in accordance with the manufacturer’s protocol. After normalising the DNA, 1 ng DNA of each sample was amplified using the Power Plex^®^ 16 System (Promega, Vienna, Austria) in a 10 μl reaction. One μl of the product was mixed with Hi-Di formamide (Applied Biosystems Inc., Foster City, US) and Internal Lane Standard (ILS600), denatured and fractionated on an ABI 3730 Genetic Analyser (Applied Biosystems Inc.). The resulting data were processed and evaluated using ABI Genemapper 4.0 (Applied Biosystems Inc.).

### Immunohistochemical (IHC) and immunofluorescence (IF) stainings

#### Patient’s tumour

For the histopathological evaluation, the tumour was tested using the streptavidin-biotin peroxidase complex method with antibodies against Caldesmon (Dako, Glostrup, Denmark), S100 (Dako), CD34 (Neomarkers, Fremont, CA), Desmin, EMA, and Pan-CK (all Ventana Medical Systems, Tucson, AZ).

#### MUG-Myx2a/b morphology

For IF analysis, cells were seeded at a concentration of 1 × 10^4^ cells on polystyrene culture slides (BD Biosciences, San Diego, CA). Slides were washed with PBS and fixed by exposure to formalin 4% for 10 minutes. After drying and rehydration, the slides were treated with Large Volume UltraV-Block (ThermoScientific, Waltham, US) for 10 min at room temperature to block nonspecific binding, incubated with the primary monoclonal mouse anti-Vimentin antibody (Dako) for 30 min and, after several washing steps, incubated with the Cy2 conjugated sheep anti-mouse IgG secondary antibody (Jackson Immunoresearch, Suffolk, UK) at a dilution of 1:200 for 30 min. Nuclei were counterstained with DAPI (Invitrogen).

#### Mice tissue

IHC studies using the streptavidin-biotin peroxidase complex method were carried out on histological slides from *MUG-Myx2a* and *MUG-Myx2b* mice tumours, employing a rabbit monoclonal primary antibody against the anti-Ki-67 (clone 30–9) (Ventana Medical Systems) using the BenchMark Ultra instrument (Ventana Medical Systems). Slides were photographed using an Olympus BX51 microscope with an Olympus DP71 microscope digital camera. The stained slides were scanned digitally and positive and negative cells were quantified using the ImageScope software (ImageScope Virtual Slide, version 6.25, Aperio Technol., Vista, US). Positivity was determined by assessing the number of positive cells / number of total cells.

### Cell proliferation analysis

#### xCELLigence System

The xCELLigence RTCA-DP device from Roche Diagnostics (OLS, Bremen, Germany) was used to monitor cell proliferation in real-time. Respectively 5 × 10^3^ *MUG-Myx2a/b* cells were seeded in electronic microtiter plates (E-Plate™, OLS) and measured for 72 h with the xCELLigence system according to the instructions in the user’s manual. Cell density measurements were performed in quadruplicate with a programmed signal detection every 20 min. Data acquisition and analyses were performed with the RTCA software (version 1.2, OLS).

#### Growth behaviour

2.5 × 10^3^ *MUG-Myx2a/b* cells were seeded into 96-well microtiter plates (Brand, Voerde-Friedrichsfeld, Germany) and the CellTiter 96^®^ AQ_ueous_ Assay (Promega, Mannheim, Germany) was performed after the manufacturer’s instructions at days 1, 2, 3, and 6. The culture medium was used as a negative control.

#### Drug sensitivity assay

*MUG-Myx2a* and *MUG-Myx2b* were adjusted to a density of 3 × 10^3^ cells and incubated in 96-well microplates. The cells were exposed to various concentrations of chemotherapeutic drugs (the DNA topoisomerase II inhibitor doxorubicin hydrochloride, the histone deacetylase inhibitors inhibitor Verinostat, and the proteasome inhibitor Bortezomib; Selleckchem, Houston, TX) for 48 h. Drug sensitivity was determined by the MTS assay following the manufacturers’ instructions in quadruplicate using a photometer (Spektramax; BMG Labtech., Offenburg, Germany) at the wavelength of 490 nm. Results were expressed as the mean from three independent experiments (*n* = 3, measured in biological quadruplicates) and error bars represent the S.D.

### Real-time chemotaxis assay

Chemotaxis assays were performed in cell invasion and migration (CIM-Plate™; OLS) using an xCELLigence RTCA-DP instrument (OLS). A CIM-plate comprises two separate parts: an upper chamber with a microporous membrane (pore size = 8 mm) embedded with gold microelectrodes on its underside and a lower chamber. This migratory assay is based on a small and harmless electrical current applied between the electrodes. Cell adherence to this surface while migrating from the upper chamber toward 10% FBS as chemoattractant placed in the lower chamber is measured. The more cells accumulate on the underside of the membrane, the more the electrical current is impeded by the insulating properties of cellular plasma membranes. The overall cell-induced impedance is measured in real-time as the cell index (CI), a dimensionless parameter.

### Tumour formation

Tumourigenicity of *MUG-Myx2a/b* clones: 7 week old female nu/nu Foxn1 mice (Charles River Laboratories, Sulzfeld, Germany) were xenotransplanted with *MUG-Myx2a* (p49) and *MUG-Myx2b* (p52) cells. Respectively 5 × 10^6^ cells were suspended in 0.2 ml of serum-free medium and subcutaneously inoculated into the right and left flank of 5 mice. The mice were observed daily and the tumour growth was monitored. All animal work was done in accordance with a protocol approved by the institutional animal care and use committee at the Austrian Federal Ministry for Science and Research (BMWF) (vote 66.010/0160-II/3b/2012).

### Mutation Analysis by Next-Generation Sequencing (NGS)

Genomic DNA of *MUG-Myx2a* and *MUG-Myx2b* was isolated on a Maxwell, MDxResearch System (Promega). Highly multiplexed PCR was used to generate amplicon libraries covering the coding region of 50 genes commonly implicated in cancer (Cancer Hotspot Panel v2, Cat.Nr. 4475346; Thermo Fisher Scientific, Waltham, MA). All analyses were performed in duplicates. Libraries were prepared using the Ion AmpliSeq Library Kit 2.0 (Cat.Nr. 4475345; Thermo Fisher Scientific) and sequencing was performed on an Ion Proton Sequencer (Thermo Fisher Scientific). Emulsion PCR and sequencing runs were performed with the appropriate kits (Ion One Touch Template Kit version 2 and Ion Proton 200 Sequencing Kit; Thermo Fisher Scientific) using Ion PI chips. Sequencing length was set to 520 flows and yielded reads ranging from 70 to 150 bp, consistent with the expected amplicon size range.

### NGS Data Analysis

Initial data analysis was performed using the Ion Torrent Suite Software version 4.1 Plug-ins (Thermo Fisher Scientific, open source, general public license). Briefly, this included base calling, alignment to the reference genome (HG19) using the Torrent Mapper and variant calling by a modified diBayes approach by taking into account the flow space information. All called variants were annotated using open source software (Annovar^34^, http://www.openbioinformatics.org/annovar/annovar download form.php, and SnpEff^35^, http://snpeff.sourceforge.net/download.html; both last accessed September 28, 2015). Coding, nonsynonymous mutation calls present in both duplicate analyses were further evaluated and visually inspected in Integrative Genomics Viewer. Variant calls resulting from technical read errors or sequence effects were excluded from the analysis. Mutations were subsequently assessed for biological relevance by computerized prediction (*in silico* prediction). Sorting Intolerant From Tolerant algorithm (SIFT) was used to generate SIFT scores.

### Low density whole genome copy number variation (CNV)

NGS libraries werer prepared using 500 ng genomic DNA and the Ion Plus Fragment Library Kit (Cat.Nr. 4471252, Thermo Fisher Scientific). DNA libraries were sequenced on the Ion Proton Sequencer yielding 8–10 million reads per library. CNV analysis was performed using the CNAnorm Bioconductor R package^36^. Briefly, reads were aligned to hg19 and summarized in windows of 10 kB size. CNAnorm was used to normalize for GC content and perform CNV analysis using coverage data from a non neoplastic reference sample. Circos plots were created using the circos software package^37^ (http://circos.ca/). Ploidy analysis of single genes was calculated in R using gene coordinates from ensembl database and ploidy ratio information from CNAnorm.

### Statistical analysis

The outcome variables were expressed as mean ± SD. The student’s unpaired *t*-test and the exact Wilcoxon’s test were used to evaluate differences between groups with the PASW statistics 18 software (IBM Corporation, Somers, NY). Two-tailed *P*-values below 0.05 were considered statistically significant. Graphic data were prepared with SigmaPlot^®^ (Systat Software Inc., San Jose, US).

## Additional Information

**How to cite this article**: Lohberger, B. *et al*. Establishment of a novel cellular model for myxofibrosarcoma heterogeneity. *Sci. Rep.* **7**, 44700; doi: 10.1038/srep44700 (2017).

**Publisher's note:** Springer Nature remains neutral with regard to jurisdictional claims in published maps and institutional affiliations.

## Figures and Tables

**Table 1:** STR genotype of the patient primary tumour, the *MUG-Myx2a* (p45) and the *MUG-Myx2b* (p48) cell lines.

STR-Locus	Patient tissue	*MUG-Myx2a*	*MUG-Myx2b*
D3S1358	15,16	16,17	16,17
TH01	9	6,9	6,9
D21S11	29	29	29
D18S51	14,16	14	13,14
Penta E	5,9	5,9	5,9
D5S818	10,11	10,11	10,11
D13S317	10,11	11	11
D7S820	10,11	10,11	10,11
D16S539	12,13,14	13,14,15	13,14
CSF1PO	12	12,14,15	11,12
Penta D	11	11	11
Amelogenin	X	X	X
vWA	17,18, 20	17,18	17,18
D8S1179	15	15	15
TPOX	8,10	8,10	8,10
FGA	20,21	21	21

**Table 2:** NGS mutation analysis in the patient primary tumour versus *MUG-Myx2a* and *MUG-Myx2b* cell lines.

Patient tissue MAF %	*MUG- Myx2a* MAF %	*MUG- Myx2b* MAF %	Gene symbol	Mutation	Transcript	Genomic coordinate	SIFT score
62.25	59.75	54.18	FGFR3	p.F386L	NM_001163213	Chr 4, 1806131	0.47
60.91	68.67	73.38	KIT	p.M541L	NM_000222	Chr 4, 55593464	0.6
58.68	69.24	50.91	KDR	p.Q472H	NM_002253	Chr 4, 55972974	0.35
0	24.56	0	PTEN	p.R173H	NM_000314	Chr 10, 89711900	0
73.88	100	100	TP53	p.R213Q	NM_000546	Chr 17, 7578211	0

MAF = minor allele frequency.

**Table 3:** CNV analysis of 50 cancer genes in the patient primary tumour versus *MUG-Myx2a* and *MUG-Myx2b* cell lines.

Gene symbol	Chromosome	Tumor tissue	*MUG-Myx2a*	*MUG-Myx2b*
**ABL1**	chr9	0.9192	0.9438	1.0374
**AKT1**	chr14	0.4480	1.0000	1.3372
**ALK**	chr2	1.1441	1.6414	1.6716
**APC**	chr5	1.0856	0.9466	0.9630
**ATM**	chr11	0.8256	0.8087	0.9741
**BRAF**	chr7	1.0480	1.5278	1.5718
**CDH1**	chr16	1.0275	0.8986	0.8811
**CDKN2A**	chr9	0.1051	NA	NA
**CSF1R**	chr5	0.8576	0.8666	0.8804
**CTNNB1**	chr3	0.9304	1.3203	1.4344
**EGFR**	chr7	1.3430	1.4048	1.6639
**ERBB2**	chr17	1.0767	1.1440	1.5046
**ERBB4**	chr2	1.0075	1.1100	1.0886
**EZH2**	chr7	1.0183	0.9342	1.0099
**FBXW7**	chr4	0.9910	1.0970	0.8166
**FGFR1**	chr8	2.2310	2.6211	2.3104
**FGFR2**	chr10	0.9797	1.1420	0.8278
**FGFR3**	chr4	1.0820	0.5976	0.9027
**FLT3**	chr13	0.8371	0.9192	1.3178
**GNA11**	chr19	1.1316	1.1151	1.6261
**GNAQ**	chr9	0.9312	0.9096	1.2227
**GNAS**	chr20	0.7208	1.2971	1.5973
**HNF1A**	chr12	0.8146	1.0399	1.4925
**HRAS**	chr11	1.5547	1.4537	1.1087
**IDH1**	chr2	1.2772	0.7513	0.8040
**IDH2**	chr15	0.9802	1.0735	1.3360
**JAK2**	chr9	0.8101	1.1047	0.8251
**JAK3**	chr19	1.0752	2.0590	2.0163
**KDR**	chr4	0.7415	0.5758	0.9654
**KIT**	chr4	1.0669	0.8604	1.1048
**KRAS**	chr12	1.2703	2.1689	1.4992
**MET**	chr7	1.3360	1.7628	1.6280
**MLH1**	chr3	0.1939	NA	NA
**MPL**	chr1	0.7063	0.9830	1.3276
**NOTCH1**	chr9	1.1330	1.2689	1.4291
**NPM1**	chr5	0.7543	1.1765	0.9742
**NRAS**	chr1	1.0851	2.2651	2.3976
**PDGFRA**	chr4	1.2816	1.1329	1.1979
**PIK3CA**	chr3	1.1218	1.0355	0.7826
**PTEN**	chr10	1.0146	1.0984	0.7667
**PTPN11**	chr12	0.9374	1.1896	1.4320
**RB1**	chr13	0.7520	0.7366	1.0491
**RET**	chr10	0.7390	1.0544	1.1011
**SMAD4**	chr18	1.0836	1.4800	1.3592
**SMARCB1**	chr22	1.1673	1.3899	1.6396
**SMO**	chr7	1.4613	1.3363	1.8677
**SRC**	chr20	1.1173	1.1082	1.4421
**STK11**	chr19	1.6188	1.6498	1.7545
**TP53**	chr17	1.2487	1.7442	1.8102
**VHL**	chr3	0.5489	0.6544	0.4328
